# Lung function development after preterm birth in relation to severity of Bronchopulmonary dysplasia

**DOI:** 10.1186/s12890-017-0441-3

**Published:** 2017-06-30

**Authors:** Petra Um-Bergström, Jenny Hallberg, Per Thunqvist, Eva Berggren-Broström, Martin Anderson, Gunilla Adenfelt, Gunnar Lilja, Giovanni Ferrara, C. Magnus Sköld, Erik Melén

**Affiliations:** 1 Department of Pediatrics, Södersjukhuset, Sachs’ Children and Youth Hospital, 118 83 Stockholm, Sweden; 2 Lung Allergy Clinic, Karolinska University Hospital, Stockholm, Sweden; 3 Department of Medicine Solna, Karolinska Institutet, Stockholm, Sweden; 4 Institute of Environmental Medicine, Karolinska Institutet, Stockholm, Sweden; 5 Department of Clinical Science and Education, Södersjukhuset, Karolinska Institutet, Stockholm, Sweden; 6 Department of Laboratory Medicine, Clinical Physiology, Karolinska Institutet, Stockholm, Sweden; 7 Department of Medical Sciences, Occupational and Environmental Medicine, Uppsala University, Uppsala, Sweden; 8 Centre for Occupational and Environmental Medicine, Stockholm County Council, Stockholm, Sweden

**Keywords:** Adolescents, Bronchopulmonary dysplasia, Lung function tests, Oscillometry, Spirometry, Ergospirometry

## Abstract

**Background:**

Bronchopulmonary dysplasia (BPD) is a strong risk factor for respiratory morbidity in children born preterm. Our aims were to evaluate lung function in adolescents born preterm with and without a history of BPD, and to assess lung function change over time from school age.

**Methods:**

Fifty-one individuals born in Stockholm, Sweden between gestational ages 24 to 31 weeks (23 neonatally diagnosed with respiratory distress syndrome (RDS) but not BPD, and 28 graded as mild (*n* = 17), moderate (*n* = 7) or severe (*n* = 4) BPD) were examined in adolescence (13–17 years of age) using spirometry, impulse oscillometry (IOS), plethysmography, and ergospirometry. Comparison with lung function data from school age (6–8 years of age) was also performed.

**Results:**

Adolescents with a history of BPD had lower forced expiratory volume in 1 s (FEV_1_) compared to those without BPD (−0.61 vs.-0.02 *z-scores, P* < 0.05), with lower FEV_1_ values significantly associated with BPD severity (*P* for trend 0.002). Subjects with severe BPD had higher frequency dependence of resistance, R_5–20_, (*P* < 0.001 vs. non-BPD subjects) which is an IOS indicator of peripheral airway involvement. Between school age and adolescence, FEV_1_/FVC *z-scores* decreased in all groups and particularly in the severe BPD group (from −1.68 *z-scores* at 6–8 years to −2.74 *z-scores* at 13–17 years, *p* < 0.05 compared to the non-BPD group).

**Conclusions:**

Our results of spirometry and IOS measures in the BPD groups compared to the non-BPD group suggest airway obstruction including involvement of peripheral airways. The longitudinal result of a decrease in FEV_1_/FVC in the group with severe BPD might implicate a route towards chronic airway obstruction in adulthood.

## Background

In the last decades, an increasing number of infants born at gestational ages (GA) less than 32 weeks survives thanks to improved neonatal care including the use of surfactant, antenatal steroids, and more gentle ventilatory support [1].

Due to preterm birth, lung development is interrupted during the canalicular and saccular/early alveolar phases of normal lung maturation, a process that is supposed to take place in utero*.* Perinatal exposure to inflammation, infection, mechanical ventilation, and hyperoxia may lead to further insult to the immature lung [2, 3]. Respiratory distress syndrome (RDS) is caused by insufficient levels of surfactant in the alveolus and is common in infants born preterm [4]. The definition is based on the presence of respiratory distress, increasing need for supplemental oxygen and typical chest X-ray findings without any evidence of other causes [4, 5].

Some of the infants with RDS at birth will eventually develop Bronchopulmonary dysplasia (BPD). The “old” BPD defined by Northway et al. [6] was characterized by inflammation, airway smooth muscle hypertrophy, emphysema, and parenchymal fibrosis caused by high oxygen concentration and high ventilation pressures. The success of modern neonatal care where more immature infants survive has been accompanied by the development of a new BPD phenotype with an altered disease pathogenesis compared to “old” BPD. The “new” BPD is characterized by even more immature lung tissue affected by reparative processes, impaired alveolarization, and dysmorphic vascular growth [7, 8]. BPD is currently defined by the need for supplemental oxygen at 28 days of age and can be further classified as moderate or severe BPD based on the level of oxygen need at 36 weeks of gestation [9]. Although previous long-term follow-up studies have shown a negative association between BPD and lung function [10, 11], as well as an increased risk of developing chronic airway obstruction later in adulthood [12–14], long-term outcome studies are in need of constant update thanks to the rapid advances in neonatal care. Further, few studies have addressed longitudinal changes up to adolescence, or assessed small airway function, in relation to *severity* of BPD in this group of patients.

We hypothesized that severity of BPD in children born preterm is associated with impairment of several aspects of lung function that persists into adolescence. The primary aim was therefore to extensively evaluate the influence of BPD severity on exercise capacity and lung function assessed by static and dynamic spirometry, and impulse oscillometry, in a cohort of adolescents born preterm. In addition, we aimed to assess change of lung function from 7 to 14 years of age in relation to BPD severity.

## Methods

### Participants

The study group consisted of 51 out of 60 children born before 32 weeks of GA as previously described in detail [15]. All children had been treated at the Neonatal Unit of Sachs’ Children’s Hospital, Stockholm, Sweden, between 1992 and 1997. Twenty-eight had been diagnosed with BPD and the remaining 23 with RDS, but not BPD (non-BPD).

The diagnosis of BPD was based on the need for supplementary oxygen at 28 days of age. The severity of BPD was determined at 36 weeks GA as follows: 1) mild BPD - breathing air; 2) moderate BPD - need for supplemental oxygen <30%; 3) severe BPD ≥30% supplemental oxygen and/or continuous positive airway pressure (CPAP) or ventilator [9].

Perinatal and neonatal data were obtained from medical records and included treatment with prenatal and postnatal steroids, GA at birth, birth weight (BW), instillation of surfactant, number of days on ventilator, CPAP, supplemental oxygen, retinopathy of prematurity (ROP), persistent ductus arteriosus (PDA), necrotizing enterocolitis (NEC), and septicemia. Information on parental smoking was retrieved from questionnaires answered by the parents when the children were approximately 7 years old [15].

### Spirometry

At mean age14.5 years (range 13.2–17.0), hereafter referred to as adolescence, lung function in terms of dynamic spirometry and static lung volumes was measured according to American Thoracic Society criteria, using the Sensormedics 6200 body plethysmograph (SensorMedics; Yorba Linda, CA, USA) [16]. Examination was performed with the subject in sitting position and wearing a nose clip. Each subject performed at least three acceptable slow and forced vital capacity expirations. The highest values of forced vital capacity (FVC), and forced expiratory flow in 1 s (FEV_1_), in addition to mean values of total lung capacity (TLC), functional residual capacity (FRC) and residual volume (RV) were registered. Spirometry at 6–8 years of age has been previously described [15].

### Impulse oscillometry (IOS)

The data retrieved from the IOS measurements is thought to represent complex functions of the lung such as small airway obstruction and airway mechanics, including the elastic properties of the lung [17, 18]. Testing was performed using the Jaeger MasterScreen-IOS system (Carefusion Technologies, San Diego, CA, USA). The method has been described in detail elsewhere [19, 20]. In short, pressure impulses were sent from a loudspeaker through the respiratory system. The subjects were encouraged to breathe tidal breathing with the lips tightly sealed around the mouthpiece and supporting cheeks with their hands to avoid impulse pressure loss due to upper airway shunt. After quality inspection, the mean value of resistance at 5 and 20 Hz (R_5_, R_20_), frequency dependence of resistance (R_5–20_) and the area of reactance (AX) were used for analysis [20–23]. IOS testing at 6–8 years of age has been previously described [15].

### Ergospirometry

Maximal exercise capacity was measured using an incremental Monark cycle ergometer (Electronic Ergomedic 839E, Monark Exercise AB, Vansbro, Sweden). Subjects used a mouthpiece and wore a nose clip. Heart rate was monitored continuously. Minute ventilation, oxygen output (VO_2_) and carbon dioxide output were measured and calculated from a mixing chamber every 30 s using a SensorMedics Vmax Encore (SensorMedics, Yorba Linda, CA, USA). Subjects were instructed to begin pedaling at 60–70 revolutions/min. After 3 min of unloading cycling, load was increased every minute by 15 W. The participants were encouraged to cycle as long as possible. Peak values for all variables were obtained by averaging data over the last 20 s of maximum completed work. Peak VO_2_ in ml/kg/min was predicted using formulae for healthy subjects [24–26].

### Statistical analysis

Demographic data are presented as median and range for continuous variables, or numbers and percentages for categorical variables. Due to non-normally distributed data, comparisons between groups were performed using the Wilcoxon rank-sum test for continuous variables. The Pearson’s χ-squared test was used for categorical outcomes.

FVC, FEV_1_ and FEV_1_/FVC were converted to z-scores using the Global Lung Initiative reference values (GLI) [27]. Cross-sectional and longitudinal comparisons between groups were made using the Wilcoxon rank-sum test. Trends across BPD severity groups were assessed using the nonparametric test developed by Cuzick for trend across ordered group [28].

Associations between other lung function outcomes and BPD severity groups were analyzed using linear regression on the median [17, 29–31], adjusting for sex, height, and age when appropriate.

The influence of gestational age, treatment with prenatal and postnatal steroids, surfactant, ROP, PDA, necrotizing enterocolitis, septicemia and maternal smoking on the relationship between BPD and lung function outcomes was evaluated with linear regression on the median.

For longitudinal analysis of impulse oscillometry data mixed models was used. Time-dependent covariates included in the model were height and age. BPD group and sex were time-invariant covariates. To assess potential variations of the effect of BPD on lung function over time, an interaction term between time and BPD group was included in the model. *P*-values of <0.05 were considered statistically significant. Analyses were performed with the Stata 13.1 software package (StataCorp LP, College Station, TX, USA).

## Results

### Patient characteristics

**Fig. 1:**
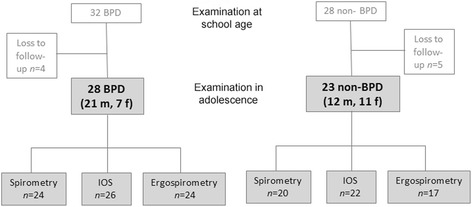
Schematic description of the patient cohort. Abbreviations: BPD = Bronchopulmonary Dysplasia; IOS = Impulse Oscillometry; m = male; f = female

Of the original 60 participants, 51 (85%) were included in the follow-up at adolescence. Twenty-eight had been diagnosed with BPD (mild *n* = 17; moderate *n* = 7; severe *n* = 4) and 23 had a neonatal diagnosis of RDS, but not BPD (non-BPD), Fig. 1. Perinatal data of all the participants are presented in Table 1.

**Table 1:** Perinatal characteristics of study participants born preterm, by BPD severity

	Mild BPD	Moderate BPD	Severe BPD	All BPD	Non-BPD	*P*-values
	*n* = 17	*n* = 7	*n* = 4	*n* = 28	*n =* 23	(All vs Non BPD)
Gestational age at birth, weeks	27 (24–30)	28 (25–30)	28 (25–29)	27 (24–30)	30 (28–31)	<0.001
Birth weight, g	995 (654–1520)	1145 (597–1252)	905 (775–1210)	1003 (597–1520)	1425 (845–2094)	0.001
Male sex	12 (71%)	6 (86%)	2 (50%)	21 (75%)	12 (52%)	0.51
Instillation of surfactant	1 (5.9%)	3 (43%)	2 (50%)	6 (21%)	1 (4.3%)	0.24
Ventilation therapy, days	0 (0–34)	4 (0–38)	23 (0–33)	1 (0–38)	0 (0–5)	0.003
CPAP, days	31 (4–70)	32 (3–55)	38.5 (13–60)	32 (3–70)	4 (0–18)	<0.001
Supplemental O_2_, days	54 (27–83)	72 (28–96)	141 (105–180)	62 (27–180)	3 (0–26)	<0.001
Maternal smoking during pregnancy	2 (12%)	2 (29%)	4 (100%)	8 (29%)	6 (26%)	0.06
Prenatal corticosteroid therapy	10 (60%)	4 (57%)	2 (50%)	16 (57%)	15 (65%)	0.96
Septicemia	9 (53%)	4 (57%)	3 (75%)	16 (57%)	4 (17%)	0.22
PDA	9 (53%)	5 (71%)	2 (50%)	16 (57%)	7 (30%)	0.36
ROP grade 3–4	5 (29%)	3 (43%)	2 (50%)	10 (36%)	1 (4.3%)	0.007

Abbreviations: *BPD* Bronchopulmonary dysplasia, *CPAP* continuous positive airway pressure, *PDA* patent ductus arteriosus, *ROP* retinopathy of prematurity

Data are presented as median (range) or numbers (%)

### Spirometry

There was a negative trend for FEV_1_ and FEV_1_/FVC measured in adolescence associated with BPD severity (*P* trend 0.002 for FEV_1_ and 0.001 for FEV_1_/FVC). Comparing BPD severity groups to the non-BPD group, significantly lower values for FEV_1_, FVC and FEV_1_/FVC was seen in the severe BPD group (Table 2). There were no significant differences between groups for TLC or RV (Table 3).

**Table 2:** Expiratory flows and volumes in z-scores in school children and adolescents with and without history of BPD

	Mild BPD	Moderate BPD	Severe BPD	All BPD	Non-BPD	P trend
	*n* = 13	*n* = 7	*n* = 4	*n* = 24	*n* = 20	
Spirometry at 6–8 years						
FEV_1_ *z-scores*	−1.10** (−1.63;-0.12)	−0.63* (−1.38;-0.41)	−2.63** (−3.56;-2.33)	−1.35 *** (−2.11;-0.41)	−0.13 (−0.51;-0.58)	<0.001
FVC *z-scores*	−1.09** (−1.50;-0.12)	−0.54 (−0.62;-0.02)	−2.21** (−2.92;-1.50)	−0.82*** (−1.50;0.03)	0.22 (1.18;0.70)	0.001
FEV_1_/FVC *z-scores*	−0.05 (−1.02;0.90)	−0.26 (−2.03;0.05)	−1.90* (−2.34;-1.04)	−0.26 (−1.70;-0.16)	−0.03 (−1.23;0.59)	0.022
Spirometry at13–17 years						
FEV_1_ *z-scores*	−0.40 (−1.48;-0.10)	−0.62 (−2.03;0.38)	−2.43** (−2.95;-2.14)	−0.61* (−2.04;0.07)	−0.02 (−0.37;0.55)	0.002
FVC *z-scores*	−0.31 (−0.96;0.28)	0.29 (0.00;1.63)	- 0.77* (−1.28;-0.33)	- 0.18 (−0.85;0.28)	0.35 (−0.43;0.66)	0.175
FEV_1_/FVC *z-scores*	−0.18 (−0.94;0.12)	−1.04 (−2.80;-0.22)	−2.95** (−3.20;-2.17)	−0.82* (−2.57;-0.10)	−0.47 (−1.09;0.58)	0.001

Comparing between non-BPD and BPD groups using the Wilcoxon rank-sum test. *P* * ≤0.05, ** ≤ 0.01, *** ≤0.001. *P*-values for trend using nonparametric test for trend across ordered groups. Trend test comparing Non-BPD vs mild, moderate, and severe BPD

Data expressed as median (25th and 75th centiles)

**Table 3:** Difference in adjusted medians of lung function when comparing BPD groups to “no BPD”

	Mild BPD	Moderate BPD	Severe BPD	All BPD	Non-BPD	P trend
Static spirometry^a^	*n* = 15	*n* = 7	*n* = 4	*n* = 26	*n* = 20	
TLC (mL)	−464 (−1151;224)	345 (−460;1150)	18 (−938;974)	−373 (−932;186)	Ref	0.848
RV (mL)	−148 (−536;240)	−147 (−602;307)	215 (−325;754)	−100 (−360;160)	Ref	0.773
Impulse oscillometry^a^	*n* = 16	*n* = 7	*n* = 3	*n* = 26	*n* = 22	
R_5–20_ (kPa/L/s)	0.036 (−0.019;0.090)	0.013 (−0.069;0.095)	0.340*** (0.218;0.461)	0.041 (−0.010;0.093)	Ref	0.029
AX (kPa/L)	0.034 (−0.322;0.38)	0.134 (−0.403;0.670)	3.704*** (2.907;4.502)	0.115 (−0.187;0.418)	Ref	0.275
Ergospirometry^b^	*n* = 13	*n* = 7	*n* = 4	*n* = 24	*n* = 17	
VO_2_ (mL/kg/min)	−1.7 (−9.3;5.9)	−2.3 (−11.8;7.2)	−9.1 (−20.5;2.3)	−4.0 (−10.9;2.9)	Ref	0.069

^a^Analysis with linear regression on the median adjusting for age, sex and height. ^b^Analysis with linear regression on the median adjusting for sex. Trend test comparing Non-BPD vs mild, moderate, and severe BPD

Comparing between non-BPD and BPD groups including all BPD using (a) and (b). *P* * ≤0.05, ** ≤ 0.01, *** ≤0.001

Data presented as difference (95% CI)

### Impulse oscillometry (IOS)

Frequency dependence of resistance (R_5–20_) showed a trend of increasing resistance values with BPD severity (*P* trend 0,029), Table 3. The adolescents with severe BPD had significantly higher adjusted R_5–20_ (0.34 kPa/L/s 95% CI 0.22; 0.46, *P* < 0.001) and higher AX (3.70 kPa/L 95% CI 2.91; 4.50, *P* < 0.001) compared to non-BPD. The groups with mild and moderate BPD did not differ in comparison with non-BPD.

### Ergospirometry

There were no significant differences in VO_2_ between non-BPD and BPD or within the different groups of BPD (Table 3).

### Longitudinal comparison of spirometry and IOS measures between 7 and 14 years of age

**Fig. 2:**
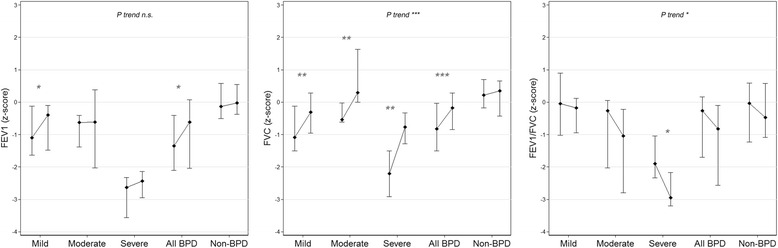
Z-scores presented as median (25th and 75th centiles) for FVC, FEV_1_ and FEV_1_/FVC at 7 and 14 years of age in children with no, mild, moderate, severe and all BPD. *P*-values for comparison of the change from 7 to 14 years in mild, moderate and severe BPD groups compared to the non-BPD group. Nonparametric test for trend across ordered groups comparing mild, moderate and severe BPD to the non-BPD group. *P* * ≤0.05, ** ≤ 0.01, *** ≤0.001

FVC and FEV_1_ z-scores increased over time in all groups (Table 2, Fig. 2). For FVC, the increase was significantly greater for all BPD groups compared to the non-BPD group, with a positive trend with increasing disease severity (*P* < 0.01 to *P* < 0.001, *P* trend <0.001). For FEV_1_, a significantly larger increase was only observed for the overall BPD (*P* = 0.02) and mild BPD (*P* = 0.003) groups, compared with the non-BPD group. In contrast, FEV_1_/FVC z-scores decreased over time in all groups (with *P trend* < 0.05 in relation to BPD severity). Comparing between groups, the FEV_1_/FVC decrease over time was overall larger in all BPD groups compared to the non-BPD group (borderline significant, *P* = 0.059) and in particular comparing severe BPD and non-BPD (*P* < 0.05).

For IOS, a larger increase of R_5–20_ values was observed over time in the severe BPD group compared to the non-BPD group (beta +0.011 kPa/L/s 95%CI 0.033; 0.253, *P* = 0.011), while no differences were seen for the other BPD groups.

### Proportion of individuals below the lower limit of normal

In the severe BPD group, 75% (*n* = 3) of the individuals had a FEV_1_/FVC z-score below the lower limit of normal (−1.64 z-scores) both at age 7 and in adolescence. Corresponding numbers were 19% (*n* = 3) at age 7 and 15% (*n* = 2) in adolescence for mild BPD, and 43% (*n* = 3) at both ages for moderate BPD.

## Discussion

In the current study, we found a trend towards more severe airway obstruction measured by spirometry and IOS with increasing BPD severity. This extends and confirms the findings of both older [11] and more recent [32] cohorts of individuals born preterm. Longitudinal assessment of spirometry suggest a pattern of increasing airway obstruction over time in subjects with a history of BPD. While FVC increased significantly more in all BPD groups compared to the non-BPD group, only marginal increase over time was observed for FEV_1_. Consequently, a pattern of decreased FEV_1_/FVC development over time was observed, with a significant trend test in relation to BPD severity and thus the largest decrease in the severe BPD group compared to non-BPD. It should be noted that borderline significant differences in FEV_1_/FVC development between BPD and non-BPD groups were observed *P* = 0.059), presumably due to a small size in our study.

Few studies have reported longitudinal data on lung function tested in individuals born preterm, and the results are diverging. Vollsæter et al. [14] reported that trajectories of the lung function indices FEV_1_ and mid-expiratory flow (FEF_25–75_) were similar from mid-childhood to adulthood in groups of individuals born preterm and term. However, airway obstruction was observed for preterm subjects during the whole study period and mostly pronounced in the group with BPD. Kotecha et al. [33] suggested tracking of spirometry measurements from 8 to 9 to 14–17 years of age in preterms born in GA 25–32 weeks, while Narang et al. [34] reported an improvement in FEV_1,_ FVC and FEF_25–75_ when comparing mid-childhood to adulthood data in ex-preterm subjects born in the pre-surfactant era.

In a younger age group of children with moderate to severe BPD, Filippone et al. [35] reported lack of lung function catch-up between early childhood and school-age. Decreasing FEV_1_/FVC from 8 to 18 years of age in children born preterm with BPD was shown by Fortuna et al. [36] and Doyle et al. [10], and between school age and adolescence in children born moderate to late preterm (at 32–36 GA) by Thunqvist et al. [17]. Nevertheless, the clinical relevance of this observation remains to be evaluated.

Possible explanations of diverse results between studies could be that children are included at different GA, and that the neonatal care has changed regarding treatment with antenatal steroids, surfactant and mechanical ventilation. Another possibility is that there might be differences in how to define the diagnosis of BPD. Many studies use criteria according to Jobe and Bancalari [9] but there have been different strategies how to set saturation limits of what is oxygen dependency that may have influenced BPD diagnosis and severity. Many of the studies, including ours, have a limited number of participants and this might contribute to the diversity because of power issues.

The longitudinal IOS data in the present study showed an increased peripheral airway resistance over time in the group of severe BPD compared to non-BPD. This is in line with the increasing obstructive pattern shown by spirometry. The lack of corresponding response in the reactance parameter Ax could be a reflection of the relative increase in FVC over time seen in the groups with BPD. To our knowledge, there are no other studies reporting IOS measured at more than one time point in individuals born preterm. Malmberg et al. [37] reported higher respiratory resistance and lower reactance measured by forced oscillation technique (FOT) in school age children with BPD. Similar results were demonstrated in pre-school children born preterm with BPD by Vrijlandt et al. [38]. Thunqvist et al. [17] showed an increased frequency dependency of resistance (R_5–20_) and AX in male subjects born moderate to late preterm measured at 16 years of age. Taken together, ex-preterm subjects seem to have signs of persistent airway obstruction measured by different oscillation techniques, and these observations warrant further studies.

Exercise capacity measured by ergospirometry is not widely described for this group of patients. We did not see any significant differences between the groups of BPD compared to non-BPD even if a tendency towards less work capacity was seen with increasing severity of BPD. In agreement with our study, Vrijlandt et al. [26] showed comparable results using ergospirometry examining exercise capacity in 19–20 years old preterm subjects and term born controls. Other studies have used incremental maximal treadmill exercise test to evaluate exercise capacity after preterm birth and modestly reduced exercise capacity in preterm subjects (but unrelated to BPD severity) has been observed in adolescents [39]. Lovering et al. [25] examined exercise capacity with exercise flow-volume loop protocol and found a reduced inspiratory reserve during near-maximal exercise in adults born preterm with and without BPD compared to term born controls. They also found a more pronounced dyspnea and leg discomfort in the same groups, as well as significantly increased expiratory flow limitation during exercise in subjects with BPD.

The mechanisms underlying long-term respiratory consequences in ex-preterm individuals are being subject to extensive research. Inflammatory processes and disturbed vascularization, partly due to mechanical ventilation and supplemental oxygen, are likely to contribute to irreversible damage of the immature lung parenchyma and the small airways [40]. Animal models have been valuable in providing detailed information about lung development and how different factors may affect the immature lung [41]. For example, the preterm lamb model has proven very useful for studies on pulmonary injury related to different ventilation strategies [42]. Histologically, BPD may lead to simplified and enlarged alveolus, and these anatomical changes are believed to result from an impairment of the postnatal alveolarization process [8]. In post-mortem lung biopsies from infants with a history of BPD, transforming growth factor α (TGF-α) has been found elevated. TGF-α is thought to damage peripheral airway and alveolus development, as well as inhibit pulmonary microvascular development in animal models [43, 44].

Transforming growth factor β1 (TGF-β1) also appears to be important in the development of BPD. In bronchoalveolar lavage-fluid in infants with BPD, Ichiba et al. [45] found elevated levels of TGF-β1 compared to controls. TGF-β1 suppresses alveolar epithelial cell proliferation, resulting in arrest of the alveolarization. This has also been demonstrated in animal models [46]. In other post-mortem materials of preterm infants with severe BPD Bhatt et al. [47] noted abnormal alveolar microvessels and decreased vascular endothelial growth factor (VEGF) expression. Altogether, the more simplified alveolus and disrupted vascular growth reduces the surface area for gas exchange [8].

The strength of the current study is that the individuals are extensively examined with methods covering different aspects of lung function, such as assessment of static and dynamic lung volumes by spirometry, and of smaller airways by impulse oscillometry, at two different time points on average 7 years apart. However, as the number of patients in this study is rather small the results must be interpreted with caution. The study subjects were recruited during a time period (1992 to 1997) when transition to modern neonatal care occurred, and unfortunately, no clear distinction between “old” and “new” BPD cases can be made in our study. Another weakness is the lack of a control group of healthy, full term born individuals. Although the magnitude of lung function impairment could be estimated using the GLI reference values [27], it has been reported that the GLI underestimate FEV_1_ in Swedish healthy adults and children [17, 48]. Hence, the relative decrease of spirometry measures found in this study might be underestimated. In addition, we did not have access to health records or questionnaire data to assess current respiratory symptoms, own smoking and medication use, or information of parental smoking.

## Conclusions

In conclusion, our findings of decreased FEV_1_ *z-scores*, FEV_1_/FVC *z-scores* and impaired IOS measures in the BPD groups compared to non-BPD suggest a relationship between BPD-severity and increasing airway obstruction, including involvement of the small airways. The decrease in FEV_1_/FVC and increase of frequency dependence of resistance (R_5–20_) over time in the group with severe BPD might implicate a route towards chronic airway obstruction in adulthood.

## Abbreviations

AX: Area of reactance
BPD: Bronchopulmonary dysplasia
BW: Birth weight
CPAP: Continuous positive airway pressure
FEF_25–75_: Mid-expiratory flow
FEV_1_: Forced expiratory volume at 1 s
FVC: Forced vital capacity
GA: Gestational age
GLI: Global Lung Initiative
IOS: Impulse oscillometry
NEC: Necrotizing enterocolitis
PDA: Persistant ductius arteriosus
R_5–20_: Frequency dependency of resistance
ROP: Retinopathy of prematurity
RV: Residual Volume
TLC: Total Lung Capacity
VO_2_: Oxygen consumption
